# Outcome of Different Surgical Repair Techniques for Rhegmatogenous Retinal Detachment—A Health Economic Analysis in the Split-Dalmatia County, Croatia

**DOI:** 10.3390/healthcare12060648

**Published:** 2024-03-13

**Authors:** Ivan Borjan, Ivna Pleština-Borjan, Silvia N. W. Hertzberg, Alen Siničić, Ljubo Znaor, Beáta Éva Petrovski, Goran Petrovski

**Affiliations:** 1 Department of Ophthalmology, University Hospital Center Split, 21000 Split, Croatia; ivan.borjan@kbsplit.hr (I.B.); alen.sinicic@kbsplit.hr (A.S.); lznaor@kbsplit.hr (L.Z.); 2 School of Medicine, University of Split, Šoltanska 2, 21000 Split, Croatia; ivna.plestina.borjan@mefst.hr; 3 Center for Eye Research and Innovative Diagnostics, Department of Ophthalmology, Institute for Clinical Medicine, Faculty of Medicine, University of Oslo, 0450 Oslo, Norway; s.n.w.hertzberg@medisin.uio.no (S.N.W.H.); b.e.petrovski@medisin.uio.no (B.É.P.); 4 Department of Ophthalmology, Oslo University Hospital, 0450 Oslo, Norway; 5 UKLONetwork, University St. Kliment Ohridski–Bitola, 7000 Bitola, North Macedonia

**Keywords:** retinal detachment, scleral buckling, pars plana vitrectomy, pneumoretinopexy, incidence, health economy, QALY, Croatia

## Abstract

**Purpose:** The goal of this research is to investigate the characteristics and surgical techniques for repairing rhegmatogenous retinal detachment (RRD) and their influence on anatomical and functional success of the treatment with a special emphasis on the economic costs and outcome quality-adjusted life years (QALYs) of the procedures. **Methods:** This retrospective study analyzed data from 151 patients with RRD treated at the Department of Ophthalmology, Clinical Hospital Centre Split, Croatia, in a 3-year period, using one of three surgical techniques: pneumoretinopexy (PR), scleral buckling (SB) or pars plana vitrectomy (PPV), followed-up for at least 6 months. Demographic, pre- and post-operative ophthalmic exam parameters and surgical technique used were collected accordingly. Statistical analysis of the influence of the studied parameters upon anatomical and functional outcomes was performed, as well as health economic analysis on costs and derived utilities/QALYs of these surgical methods. **Results:** Of all patients, 130 (86%) were successfully operated on, and 21 patients (14%) required another surgical procedure. No statistically significant differences in the anatomical success between the 3 surgical techniques were found. However, the functional outcome (based on the final best corrected visual acuity (BCVA)) differed significantly. Despite improvement in the final BCVA, BCVA ≥ 0.5 was achieved in only 52 (34.4%) patients. The final BCVA showed significant association with the time elapsed from the onset of RRD to the surgical intervention, pre-operative BCVA, macular- and preoperative lens- status and type of surgery. The estimated diagnosis-related group (DRG) cost for day and inpatient surgery was based upon existing DRG cost for PPV, which for PR and SB was calculated accordingly. Based upon the success of the procedure and visual outcome, the overall calculated QALYs for PR and SB appeared to be similar, while the QALYs were lower for PPV. **Conclusions:** The success rate of treating RRD mostly depends on performing an early surgical procedure (especially in the case of attached macula), identification of all retina tears and, most importantly, choosing the appropriate surgical technique. Decisions on treatment for RRD should also be based upon cost-effective and QALYs-assessed procedures, especially in countries like Croatia, where limited healthcare resources exist. This study shows PR to be efficient and most cost-effective for RRD repairment in appropriate cases.

## 1. Introduction

Retinal detachment (RD) is considered to be one of the most urgent conditions in ophthalmology, which, left untreated, will lead to permanent vision loss and blindness [1,2,3].

Rhegmatogenous retinal detachment (RRD) involves a full-thickness tear of the neurosensory retina (NSR), which is accompanied by presence of liquefied vitreous and posterior vitreous detachment (PVD) [3]. The risk of developing RRD in cases of asymptomatic tears is only 5%, whereas in symptomatic, it rises to 30–50% [2]. The most common risk factors associated with the development of RRD are high myopia, cataract surgery and eye injuries [4].

Currently, there are three surgical techniques for treating RRD, alone or as combination: pneumoretinopexy (PR), scleral buckling (SB) and pars plana vitrectomy (PPV). All of them are based on the permanent closure of the tear (whether through external or internal approaches) and the reduction or elimination of vitreoretinal traction, with or without the removal of subretinal fluid.

PR is the surgical method that has most recently been introduced and has several advantages over other surgical techniques for RRD. It is less invasive, associated with fewer complications, performed exclusively under local anesthesia and significantly more cost-effective than the other two methods [5,6]. However, PR is not a suitable choice for all cases of RRD. Traditionally, the method included less complicated cases with a smaller number of tears (not a larger span than one clock hour), limited to the upper two-thirds of the fundus and without vitreous hemorrhage [7,8,9]. Recently, PR for primary repair of RRD has been reported to result in successful anatomic outcomes in about 75% of cases, including cases with various preoperative characteristics [10]. The failure of the procedure is most associated with the failure to identify all tears or with newly occurring tears [6]. Sometimes a second surgical procedure (PPV) is needed due to development of proliferative vitreoretinopathy (PVR), which occurs in 3.3 to 10% of cases. Other complications are rare. The anatomical success rate after PR, following a single procedure, is approximately 75–80%, while the final success, with additional interventions, reaches up to 99% [7,9,10,11].

SB is the method of choice in younger, phakic patients without complete PVD, as well as in young myopes with lattice degeneration and multiple tears (especially in the lower half of the retina) in whom PVR is not present or is present in a mild form. The optical media should be transparent in order to localize the tear(s) [1]. SB results in a high rate of primary anatomical success (the retina reattaches in >90% of cases with a single surgical procedure) and significant improvement in visual acuity [11,12]. The most common cause of SB failure is PVR, which requires an additional PPV procedure. Rarely, other intraoperative complications (iatrogenic retinal tear, bleeding into the vitreous or choroidal hemorrhage) as well as postoperative complications (increased myopia, transient diplopia and strabismus due to mechanical action of the buckle, protrusion and rarely intrusion of the epiretinal buckle, infection, epiretinal macular membrane, high intraocular pressure, anterior eye segment ischemia) may occur [11,12]. SB is commonly performed under general anesthesia and requires skilled surgeons that are experienced in using indirect ophthalmoscopy.

PPV is currently the most used surgical technique in the treatment of RRD [13]. The anatomical and functional success of PPV treatment are similar to those achieved with the SB method [4,11,12,14]. The PPV method is preferred in pseudophakic patients and is indicated in eyes with multiple or large tears, posterior tears, in cases of RRD with PVR and in those with vitreous hemorrhage [15]. The most serious complications associated with PPV include iatrogenic retinal tears, iatrogenic lens damage, postoperative cataract development, PVR development and elevated intraocular pressure [4,11,12,14,16]. PPV is commonly performed under local anesthesia.

Each of the three surgical techniques for RRD has its indications and contraindications and should meet the following criteria: successful retina reattachment with a single surgical procedure achieved with minimal morbidity, performance under local anesthesia, cost-effectiveness and prevention of secondary complications that could potentially compromise vision and cause additional expenses [17].

There is no single surgical technique that can be used in all cases of RRD. Although several previous studies have analyzed the outcomes of these techniques, none of them have demonstrated a clear, significant advantage of one technique over the other [15,18,19]. However, there are very few studies comparing the cost-effectiveness among these techniques, especially when evaluating all three together [18,20,21,22,23]. To the best of our knowledge, only one recent study has compared all three techniques on a microsimulation model in terms of lifetime costs [20]. Due to rising healthcare costs, significant attention has been focused on these as important determinants in making medical decisions, especially in countries like Croatia, with limited healthcare resources.

The goal of our study is to evaluate the three contemporary surgical techniques for treating RRD by comparing their anatomical and functional outcomes and, additionally, to examine differences in the cost and outcome utility values of these surgical methods regarding their anatomical and functional outcomes.

## 2. Methods

This is a retrospective study analyzing data from 151 patients with RRD who were admitted and treated in the Eye Clinic, Clinical Hospital Centre Split, Croatia by three experienced vitreoretinal surgeons (with fifteen or more years of experience in vitreoretinal surgery), in a three-year period (January 2016 to January 2019; most consistent data from the pre-COVID-19 period). The research was conducted following the Guidelines of the Helsinki Declaration and approval by the Research Ethics Committee of the University Hospital Centre Split, Croatia.

Inclusion criteria were patients with RRD who were postoperatively followed-up for at least 6 months. The exclusion criteria were other types of RD (tractional and exudative), open globe injuries with RRD and repeated RD.

Medical records of all patients were reviewed for age, sex, refractive error, previous ophthalmological procedures, duration of retinal detachment before surgical treatment, preoperative visual acuity, involvement of fellow eye, lens status, localization and extension of RRD, macula status, presence of PVD, retinal tear characteristics (shape, size, localization and number), presence of peripheral retina degenerations and type of RRD surgical technique used. According to the surgical technique, patients were divided into three groups: PR, SB and PPV groups.

The impact of the investigated indicators (characteristics of RRD and surgical treatment methods) on the main outcome measures, which are anatomical and functional results of treatment, were evaluated accordingly. Anatomical result failure was considered to be non-attachment of the retina after one surgical procedure or re-detachment of the retina in the six-month period. Functional success from surgery was measured by the best-corrected visual acuity (BCVA) achieved six months after surgery. The patients were categorized into three groups according to their visual acuity (≤0.1, >0.1 and <0.5, ≥0.5).

Given the fact that Clinical Hospital Centre Split is the only reference hospital for residents of the Split-Dalmatia County, and the Clinic for Eye Diseases is the only place where surgical procedures for the treatment of RD are performed, we calculated the cumulative and annual incidence by age group and the total incidence per 100,000 inhabitants in the three-year monitoring period. The calculations were based on data derived from the 2011 census of Croatia, which reported a population of 454,798 inhabitants in the Split-Dalmatia County.

The present diagnosis-related group (DRG) costs of the procedures were assessed, and then we recalculated the possible DRG costs for PR, SB and phacoemulsification cataract surgery (PHACO)+PPV either as outpatient and/or inpatient procedures. The cost calculation was based upon existing DRG costs for PPV and the estimated per-minute costs for such a procedure, which were multiplied by the time associated with each procedure. Procedure durations were obtained from the literature [5,16,24]. For PHACO+PPV, the weighted costs were added to estimate the probable DRG cost assuming payment of the full costs for the PHACO part in PHACO+PPV. DRG data were obtained from the Croatian Institute of Health Insurance (https://hzzo.hr/poslovni-subjekti/hzzo-za-partnere/sifrarnici-hzzo-0 (accessed on 15 September 2023)).

Visual outcome of PR, SB and PPV was used to estimate the utility values associated with each of the surgical outcomes. The probability of each outcome path was calculated using data values and published data to systematically attach utility values to the visual outcome. Consequently, the outcome probability was multiplied by the utility values of each surgical procedure path to derive the quality-adjusted life years (QALYs) for the treatment [25,26].

The subject evaluation (proportion) was measured on a binary scale (unsuccessful/successful). The power of the study (with a two-sided z test, if the effect size is considered moderate (h = 0.5; alpha = 0.05)) required a minimum of 32 cases per group to achieve a power of 80%. If one accounts for a 20% drop out, an additional 8 patients per group should be included in the study, which means a total sample size of about 150 subjects.

The data analysis was performed using descriptive statistical analysis; number of study participants (N) and percentage (%) were presented. Data are presented in the form of line, bar and pie charts. Normality of continuous variables was tested on histogram, Q-Q plot and by the Shapiro–Wilk and Kolmogorov–Smirnov tests. In the case of non-normal distribution of continuous variables, median (Q2) and interquartile ranges (IQR: Q1–Q3) and range (minimum/maximum) were used. Due to the non-normal distribution of the continuous variables, a Mann–Whitney U test was used to detect median differences of continuous, numerical variables between the two groups. A Wilcoxon Signed Ranks test was used to analyze the visual acuity (final visual acuity, preoperative visual acuity) difference before and after the surgical procedure. Spearman’s rank correlation with correlation coefficient (rho) was used to test correlation between two ordinal variables. The Chi-square (χ^2^) and Fisher’s exact tests were used to determine the differences in the distribution of categorical variables, while a 2-sample z-test was applied to detect the differences in the proportions of the different studied groups and to compare proportion differences between the different seasons. If the sample within each column was ≤1, then the z-test could not be used. The significance level was set as *p* < 0.05 and adjusted with Bonferroni correction to *p* < 0.05/n (where n is the number of analyses). Simple logistic regression calculating odds ratios (ORs) with their 95% confidence interval (95% CI) was applied to test the association between the symptom’s duration and preoperatively identification of retinal tears and the outcome surgical procedure (successful/unsuccessful). SPSS software (SPSS version 24, IBM, Armonk, NY, USA) and Stata software (17.0 SE-Standard Edition, College Station, TX 77845, USA) were used for the statistical analyses.

## 3. Results

Overall, 151 RRD surgical procedures were performed, of which 130 (86%) were successful and 21 (14%) needed reoperation in the follow-up period.

Table 1 shows the demographic characteristics of all the patients who underwent surgery, both as a whole and in relation to the surgical outcomes.

The distribution of participants according to overall age (*p* = 0.914), sex (*p* = 0.467) and affected eye (*p* = 0.377) did not differ significantly in relation to the outcome of the procedure. The median age of the patients was 63 years (IQR: 54–70; min-max: 18–88 years) (Table 1). There were 90 (60%) males (median: 63.5 years (IQR: 55–70; min-max: 18–88)) and 61 (40%) females (median: 62 years (IQR: 53–71; min-max: 24–76)), with no significant difference in age between males and females (χ^2^ = 0.52; *p* = 0.467) (data are not shown).

**Figure 1:**
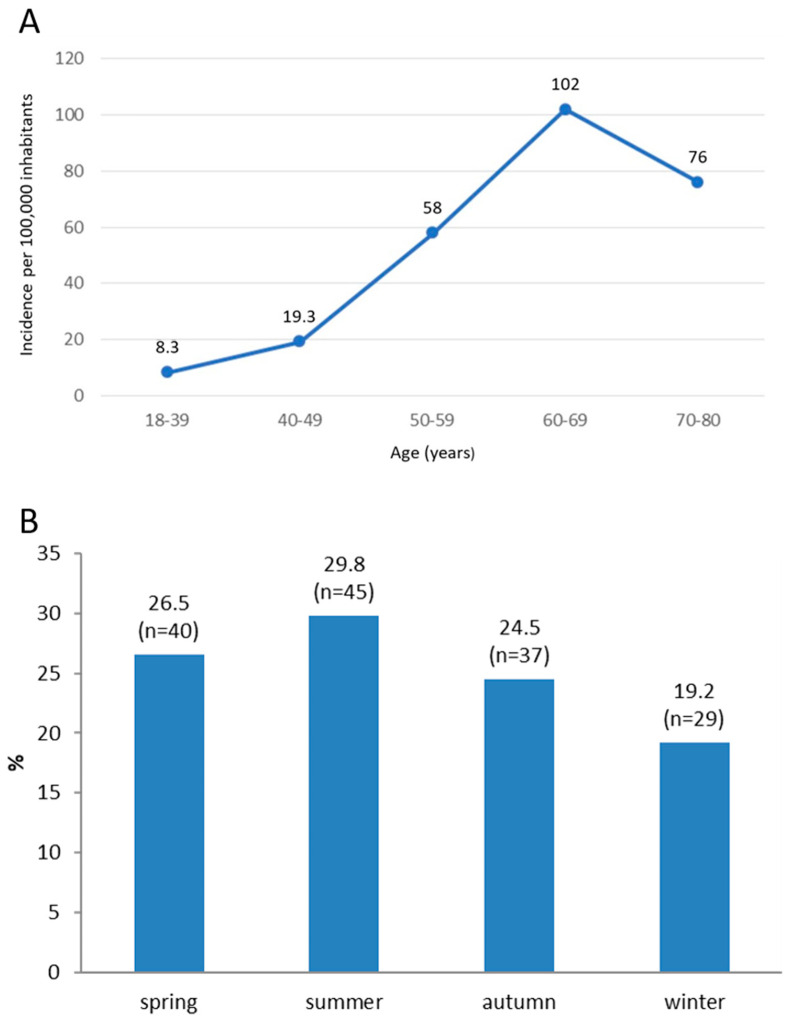
Cumulative three-year incidence of rhegmatogenous retinal detachment (RRD) in Split-Dalmatia County per 100,000 inhabitants during the period from 1 January 2016 to 1 January 2019 (**A**). Distribution of patients with RRD according to the season of disease onset (**B**).

The cumulative incidence of RRD over a three-year period per 100,000 inhabitants in the Split-Dalmatia County was 8.3 (95%CI: 3.99–15.3) for ages 18 to 39, 19.3 (95%CI: 9.9–33.7) for ages 40 to 49, 58 (95%CI: 41–80) for ages 50 to 59, 102 (95%CI: 76–135) for ages 60 to 69 and 76 (95%CI: 55–102) for ages 70 to 88 (Figure 1A). The total cumulative incidence of RRD over a three-year period in the Split-Dalmatia County was 43 (95%CI: 36–50) per 100,000 inhabitants, thus an average annual incidence was 14.3 (95%CI: 12–16.6) per 100,000 inhabitants. The seasonal distribution of RRD is shown in Figure 1B. No significant proportion differences could be detected in the seasonal distribution of RRD.

In the group of patients who had an unsuccessful surgical procedure, there were 9 (47.4%) with a symptom duration < 15 days and 10 (52.6%) > 15 days. Otherwise, in the group of patients who had a successful surgical procedure, there were 93 (75.6%) with a symptom duration < 15 days and 30 (24.4%) > 15 days (χ^2^ = 6.5; *p* = 0.011) (Table 2). The odds of an unsuccessful surgical procedure were 3.4 times higher in patients with symptoms lasting > 15 days compared to patients whose symptoms lasted < 15 days (OR = 3.4; 95%CI: 1.3–9.3).

Statistical significance was not found in the relationship between myopia (χ^2^ = 0.22; *p* = 0.640), trauma (χ^2^ = 3.02; *p* = 0.082), macular detachment (χ^2^ = 2.9; *p* = 0.090) and surgical outcome, respectively (Table 2).

In the group of unsuccessfully operated patients, retina tear was found preoperatively in 13 (65%) patients, and in 7 (35%) it was not. In contrast, in the group of successfully operated patients, retina tear was found preoperatively in 111 (86.7%) patients, and in 17 (13.3%) it was not (χ^2^ = 6.0054; *p* = 0.014) (Table 2). The odds of a successful operation, as opposed to an unsuccessful one, were 3.5 times higher in the group of patients where a tear was preoperatively found compared to the group where it was not found (OR = 3.5; 95CI: 1.2–10). Based on the prevalence of retinal tear shapes in the observed population, the horseshoe-shaped tear was most common (in 51.4% of cases, N = 76). Before the surgical procedure, a retinal tear was not found in 24 (16.2%) patients, while, in 3 patients, data on the presence or absence of retinal tear were missing. No statistically significant relationship could be found between the tear shape and surgical outcome (*p* = 0.126) (Table 2).

There was no statistically significant relationship between the lens status (phakic, pseudophakic eye; χ^2^ = 2.2; *p* = 0.136), PVD (χ^2^ = 2.3; *p* = 0.130) and peripheral degenerations (χ^2^ = 0.044; *p* = 0.834) and surgical outcome, respectively (Table 2).

Out of the 151 patients with RRD, 7 (4.6%) had previously been treated because of RRD in their fellow eye, 14 (9.3%) had lattice degeneration, 3 (2.0%) had retinal tears, while in most patients, 127 (84.1%), no peripheral degeneration was found in the fellow eye.

**Figure 2:**
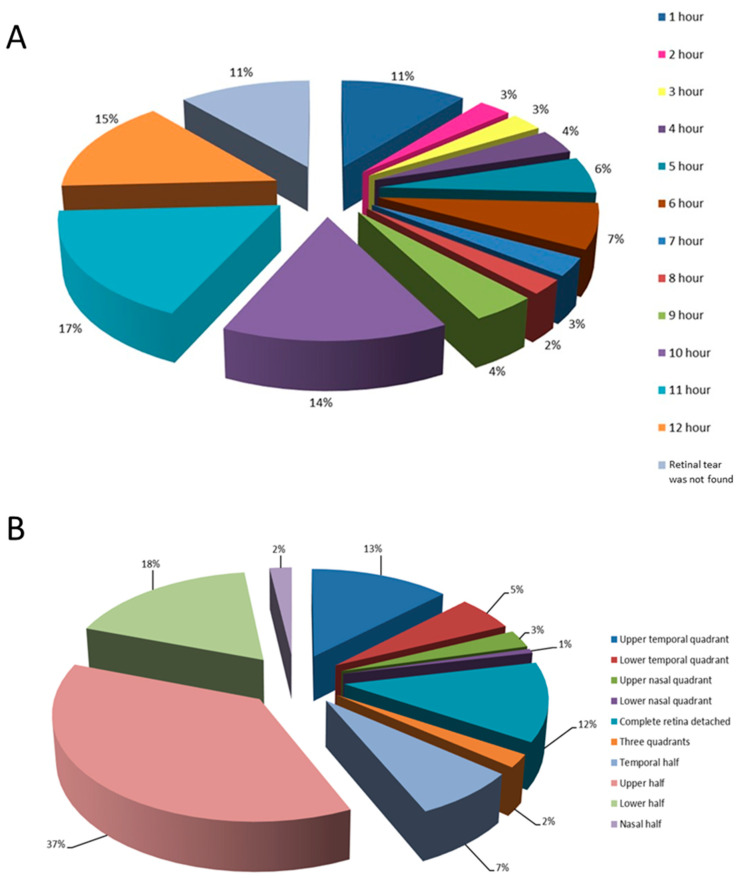
Distribution of retinal tears in the observed group of patients according to different positions on the clock-hour (**A**) and based on the location of retinal detachment (**B**).

Localizations of retinal tears and retinal detachments are shown in Figure 2A,B, respectively.

The distribution of patients according to the type of surgical procedure (χ^2^ = 1.2; *p* = 0.540) and use of combined surgical technique (PPV + PHACO) (χ^2^ = 0.979; *p* = 0.322) did not differ in relation to the surgical outcome (Table 3).

By analyzing BCVA before and after the surgical procedure, a statistically significant improvement in postoperative final BCVA compared to preoperative BCVA was observed. In 62 patients, BCVA after the procedure was better than before the procedure. The BCVA remained the same in 82 patients, while 7 patients experienced a postoperative decrease in BCVA (Z = 6.6; *p* < 0.001) (Table 4). BCVA after surgery had a statistically significant positive correlation with preoperative BCVA (rho = 0.493; *p* < 0.001).

The distribution of patients according to the duration of symptoms before surgical procedure significantly differed in relation to the categories of final BCVA (χ^2^ = 15.9; *p* = 0.043). Patients who had a surgical procedure after 30 days of RRD had the worst final BCVA (Table 5), which negatively correlated with the duration of symptoms before surgery (rho: −0.240; *p* = 0.004). The distribution of patients based on the macula status (χ^2^ = 16.4; *p* < 0.001), type of surgical procedure (χ^2^ = 46.3, *p* < 0.001) and lens status (χ^2^ = 8.03; *p* = 0.018) showed a significant relationship with the final BCVA (Table 5).

The existing DRG costs for day surgery for PPV, PHACO+PPV and PHACO were €1609.39, €1960.58 and €601.64, respectively (PR not being available as day surgery, while SB not being practiced as day surgery in Croatia). Considering the procedure times and costs per minute for day surgery, the estimated DRG cost for PR, SB, PHACO+PPV was €482.85, €2736.15, €2211.03 (PPV and PHACO already have an estimated day surgery DRG cost, which is €1609.39 and €601.64, respectively, while the new estimated cost for the PHACO+PPV procedure was assumed as full costs for the PHACO part in the combined PHACO+PPV procedure.). The estimated DRG cost for inpatient surgery for PR, SB and PHACO+PPV was €396.45, €2246.55 and €1831.01, respectively (PHACO already having an estimated inpatient DRG cost in this calculation, while PPV being assumed to have the current inpatient DRG cost for retina procedures) (Table 6).

Based on surgical outcome, probability values were calculated for the successful and unsuccessful outcome as well as for each BCVA category associated with either of the procedure’s outcome. The average utility values with respect to BCVA categories shown in Table 7 were obtained from the literature [25]. The estimated QALYs derived for PR procedures were 0.11 and 0.70 for unsuccessful and successful procedures, respectively (overall: 0.81). Similarly, for SB and PPV, the calculated QALYs for the unsuccessful and successful procedures were 0.15 and 0.67 (overall: 0.82) and 0.07 and 0.56 (overall: 0.63), respectively (Table 7).

## 4. Discussion

This study evaluates the anatomical and functional outcomes, as well as health economic aspects, of the three contemporary surgical techniques for treating RRD: PR, SB and PPV.

Our results are consistent with the findings of other studies, which report 81–92% anatomical success achieved with a single surgical procedure in less complicated cases, while in high-risk eyes, success of surgery was 65–70% [19,27,28].

Most commonly, RRD was found in individuals over 50 years of age, with the peak frequency occurring in the 60–69 age group. The 3-year incidence for this age group was nearly two times higher than that of the 50–59 age group, and five times higher than the 40–49 group; the lowest incidence was observed in the 18–39 age group. Incidence reflects the age structure of the population in the Split-Dalmatia County and risk factors associated with RRD in that age group. Among older individuals, the majority had previously undergone cataract surgery, while vitreoretinal degenerative changes and PVD were also common; thus, together with pseudophakia present, the increased risk for RRD was eminent. The average annual incidence in the Split-Dalmatia County, based on our results, is 14 per 100,000 inhabitants for the age range of 18 to 88 years, slightly higher than the European average of 10 per 100,000 inhabitants [2]. These are the first results on RRD incidence in this county, which included complete data (including phakic and pseudophakic eyes). Ivanišević et al. reported significantly lower data on the average annual incidence of RRD in the Split-Dalmatia County: 5.4 per 100,000 inhabitants (from 1989 to 1999), but their data only apply to phakic eyes [29]. Van de Put et al. reported an annual incidence in the Netherlands between 15.4 and 18.2 per 100,000 inhabitants, with peak incidence of 52.5 in the age group between 55 and 59 years [30]. Bechrakis et al. reported an annual incidence of RRD of 10 cases per 100,000 inhabitants in the European population, peaking in the 6th and 7th decades [2]. Li et al. found an annual incidence of only 8 per 100,000 inhabitants in Beijing (although the proportion of myopia individuals in this population was 66–68%) [31].

The median age of patients in our study was 63 years (range: 18–88). It is assumed that the risk of RRD is more than 20 times higher in individuals over 60 years of age compared to those under 30. Above 75 years of age, the risk slightly decreases, probably due to completion of the vitreous detachment process and time passed after cataract surgery. Other authors have also reported a significantly higher incidence of RRD in older patients [27,32,33].

In the observed patient group, there were 60% males and 40% females. Other studies have also shown a higher incidence of RRD in males compared to females: 1.3 to 2.3 times [2,32,34]. The reasons for this might be more the frequent myopia in younger males, as well as a higher prevalence of vitreoretinal adhesions and greater physical exertion [32,35].

Our study could not show a significant difference in the success of treatment based on sex. A large study from Singapore showed a three-fold higher failure rate of the surgical procedure in men compared to women. It is not clear whether this is due to any sex-specific risks or a higher rate of previous trauma [36].

RRD occurred more frequently in the summer compared to winter months, a difference which was not statistically significant, similar to reports by other authors [2,32]. Such a phenomenon could be attributed to increased outdoor physical activity during the summer months, and the influence of light and heat on the vitreous body, resulting in structural changes and heightened vitreoretinal tractions [37].

Although 37 (24%) of our patients were myopic, its presence could not affect the treatment outcome. However, myopia > 6 dioptres (D) was present in 80% of the patients under 40 years of age. Various authors have reported a four times higher risk for RRD with myopia < 3D, and ten times higher risk for 3–6D [2,32]. Considering the increase in the prevalence of myopia worldwide, a significant rise in RRD can be expected.

The process of vitreous detachment is one of the main risk factors for RRD; however, the presence of PVD in our study could not show a significant effect on the outcome of the surgical procedure. The reason for this could be the presence of PVD in 138 (91%) of the patients, which is understandable given the age of the patients. Takkar et al. reported a 50% prevalence of PVD in patients with RRD [38].

The most commonly observed type of retinal tear in the study group was horseshoe-shaped (63% cases). The tears were mostly localized in the upper quadrants of the retina and were preoperatively not found in 24 (16%) of the patients, which significantly influenced the outcome of the surgery. In patients with a preoperatively identified tear, the likelihood of the surgical procedure’s success was 3.5 times higher than in those where no tear was found. Takkar et al. provide data on 27% of preoperatively undetected tears in total and mention the most common causes of this to be lattice degeneration, complete vitreous detachment and pseudophakia [38]. In our study, pseudophakic patients had 1.9 times greater chance of having an undetected tear compared to phakic patients. In the former, tears were often small and peripherally located, making them difficult to detect. Other authors have reported similar findings [38,39,40,41,42]. Cases in which tears were not found before surgery were associated with a significantly higher surgical failure rate and a more frequent need for repeated surgical procedures, which significantly compromises the final visual outcome, thus posing further burden upon the healthcare system [38].

There was no statistically significant difference in the anatomical success of the surgical treatment between traumatic and non-traumatic RRD, probably due to the small number of traumatic RRD cases. Primary anatomical success was achieved in 27 (84.3%) out of 32 patients in the PR group, in 10 (76.9%) out of 13 patients in the SB group and in 93 (87.7%) out of 106 patients in the PPV group. Thus, surgical retreatment due to retinal re-detachment (within 6 months of follow-up) was needed in 21 (13.9%) patients. A slightly higher failure rate of the SB technique could be due to the small number of these procedures being performed in this study cohort. There were no statistically significant differences in the primary anatomical success between the surgical techniques used for treatment of RRD. Similarly, other authors have not observed statistically significant differences in the anatomical outcomes among the mentioned surgical techniques for treating RRD [43,44,45]. A large prospective study by Heimann et al. showed that in phakic patients, better anatomical success was achieved with the SB surgery method, unlike in pseudophakic eyes, where better success was achieved with PPV [15]. Comparing the success achieved with a single surgical procedure between SB and PR, Paulus et al. found higher success in the SB group (95%) compared to the PR group (67%) [46]. Although the chosen technique for the treatment of RRD could not have a statistically significant impact on the anatomical outcome in our study, it significantly influenced the functional outcome (final BCVA).

In our study, the lens status did not significantly affect the success of the surgical procedure. Out of a total of 151 patients, 57 (38%) were pseudophakic before RRD occurred. Additionally, in 33 of 106 patients who underwent PPV surgery, combined PHACO+PPV procedure was performed. In our study, RRD in pseudophakic patients was slightly more common than that reported by other authors [31]. The lens status could, however, have a significant impact on the final functional outcome, with BCVA being significantly better in the phakic eyes.

In our study, the macula was detached in 57% of the patients, which is consistent with results reported in other Western countries, ranging from 40% to 60% [32]. In contrast, in developing countries, macular detachment occurs in more than 85% of RRD [32,47]. The macula status could not significantly affect the success of the surgical procedure in our study, but it had a statistically significant impact on the postoperative BCVA. In patients with detached macula, the final BCVA was 2.1 times more likely to be ≤0.1 compared to being ≥0.5. The results indicate the need for early surgical treatment of RRD when the macula is not yet detached. According to Mahmoudi et al., in detached macula cases, a final visual acuity ≥0.5 is achieved in only 27.8% of cases, compared to those with an attached macula, where the same visual acuity could be achieved in 78% of patients [48].

The success of the surgical procedure negatively correlated with the time passed from the onset of RRD to the surgical intervention. The likelihood of surgical procedure failure was 3.4 times higher in the group of patients whose symptoms lasted for 15 days or more before surgery, compared to those whose symptoms lasted less than 15 days. Similarly, the final BCVA was significantly better in the group of subjects with a shorter duration of symptoms before surgery. A longer duration of RRD before surgery could lead to the development of PVR and a higher rate of surgical failure. Furthermore, it could result in macular detachment, which is the most common reason for poor functional outcome. Other authors also emphasized the importance of early surgical intervention for both outcomes (anatomical and functional) [27,49].

Our study also demonstrated a positive correlation between final and preoperative BCVA. Although the final BCVA was significantly better than the preoperative BCVA, good visual acuity (≥0.5) could be achieved in only 52 (34.4%) patients. Similar results have been reported by Williamson et al. as well [27].

In our study, patients treated with PR and SB achieved better final BCVA than those treated with PPV. A portion of the lower postoperative BCVA in patients treated with PPV may be attributed to the development of post-PPV cataracts. The study by Heimann et al. could not show a statistically significant difference in the final visual acuity achieved between those operated with the PPV and those with SB [15]. Paulus et al. reported significantly better visual acuity in patients treated with SB compared to PR; the former achieved a postoperative visual acuity of ≥0.5 in 89% of cases compared to 72% treated with PR [46].

PR and SB had almost the same expected QALYs in our study cohort, which was greater than the expected QALY for PPV. Our QALY results for PPV differed from others found in the literature, where PPV had the highest expected values [20,21]. Poorer functional outcomes in patients undergoing PPV, compared to other techniques, may be partly attributed to RRD severity, patient age, cataract development or silicone oil use. However, it is crucial to note that PPV was not exclusive to severe RRD or older patients in our study population, as the other two techniques also addressed some complex cases. Additionally, the assessment of final visual acuity was performed six months after the surgical procedure, in which period silicone oil was removed and cataract issues were mostly resolved. This ensures that the QALY values for PPV, based on final functional outcomes, are reliable.

Our primary focus is the patient’s well-being in choosing a surgical technique, but we also weigh cost-effectiveness. If comparable or better outcomes can be achieved at significantly lower costs, especially in resource-constrained settings, we see it as a viable option. It appears that for appropriately selected cases (RRD cases which follow general guidelines for PR, cooperative patients who can maintain post-procedure positioning, surgeons who are skilled and familiar with this procedure and are capable of solving possible postoperative complications), PR could be considered the treatment of choice for RRD due to its effectiveness, shortness and cost-saving benefits of the procedure. Furthermore, it is less invasive, could be easily performed as outpatient procedure, with no need for general anaesthesia, with fewer complications compared to two other techniques (severe infections, cataract development, diplopia, refractive error change) and most importantly, without sacrificing success rate of surgical procedure, final BCVA and excepted QALYs, which our study has demonstrated.

The retrospective nature of our study is a main limitation of this study, as well as relatively small sample size and incompleteness of certain data in the medical records.

## 5. Conclusions

Our study showed that good anatomical success of the surgical procedures for RRD treatment is not followed by equally good functional outcome. No single surgical technique can be used for all cases of RRD. Each technique has its indications and contraindications and should provide successful retina reattachment with a single surgical procedure achieved with minimal morbidity, under local anesthesia, and prevention of secondary complications that could potentially compromise vision and cause additional expenses. Moreover, the success rate of treating RRD mostly depends on the following factors: early surgical intervention (particularly in the cases with attached macula), identification of all retina tears, macular and lens status and the preoperative BCVA. In addition, decisions regarding the treatment of RRD should be based on cost-effectiveness and QALYs, especially in countries like Croatia, where limited healthcare resources exist. The results of our study indicated that, given its effectiveness, minimally invasive nature, procedure duration and cost-effectiveness, PR could be a suitable procedure of choice for treating appropriately selected cases of RRD.

## Figures and Tables

**Table 1:** Demographic characteristics of the patients and their surgical outcome.

			N (%)		
			Surgical Outcome		
		Total (N = 151)	Unsuccessful (N = 21)	Successful (N = 130)	*p*
Sex	Males	90 (59.6)	11 (52.4)	79 (60.8)	0.467
	Females	61 (40.4)	10 (47.6)	51 (39.2)	
Age	<40	10 (6.6)	1 (4.8)	9 (6.9)	0.784
	40–49	12 (7.9)	3 (14.3)	9 (6.9)	
	50–59	38 (25.2)	4 [19]	34 (26.2)	
	60–69	49 (32.5)	7 (33.3)	42 (32.3)	
	≥70	42 (27.8)	6 (28.6)	36 (27.7)	
Eye	Right	80 (53.0)	13 (61.9)	67 (51.5)	0.377
	Left	71 (47.0)	8 (38.1)	63 (48.5)	
Age (years)	Median (Q1–Q3; min-max)	63 (54–70; 18–88)	63 (58–70; 26–81)	63 (54–71; 18–88)	0.914

Q = quartile; N = number; *p* = *p*-value; *p* < 0.05.

**Table 2:** Distribution of patients according to clinical characteristics of retinal detachment and symptoms duration in relation to the surgical outcome.

			N (%)		
			Surgical Outcome		
		Total (N = 151)	Unsuccessful (N = 21)	Successful (N = 130)	*p*
Symptoms duration (days) ^1^	<15	102 (71.8)	9 (47.4)	93 (75.6)	**0.011**
	≥15	40 (28.2)	10 (52.6)	30 (24.4)	
Myopia	Yes	37 (24.5)	6 (28.6)	31 (23.9)	0.640
	No	114 (75.5)	15 (71.4)	99 (76.1)	
Trauma	Yes	9 (6.0)	3 (14.3)	6 (4.6)	0.082
	No	142 (94.0)	18 (85.7)	124 (95.4)	
Macula status ^1^	Attached	63 (42.6)	12 (60.0)	51 (39.8)	0.090
	Detached	85 (57.4)	8 (40.0)	77 (60.2)	
Retinal tear ^1^	Found	124 (83.8)	13 (65.0)	111 (86.7)	**0.014**
	Not found ^2^	24 (16.2)	7 (35.0)	17 (13.3)	
Retinal tear shape ^1^	Horseshoe	76 (51.4)	7 (35.0)	69 (53.9)	0.126
	Round	23 (15.5)	4 (20.0)	19 (14.8)	
	Horseshoe + round	17 (11.5)	1 (5.0)	16 (12.5)	
	Gigantic	4 (2.7)	1 (5.0)	3 (2.3)	
	Linear	4 (2.7)	0	4 (3.1)	
	Not found ^2^	24 (16.2)	7 (35.0)	7 (35.0)	
Lens status	Phakic	94 (62.2)	10 (47.6)	84 (64.6)	0.136
	Pseudophakic	57 (37.8)	11 (52.4)	46 (35.4)	
PVD	Yes	138 (91.4)	21 (100.0)	117 (90.0)	0.130
	No	13 (8.6)	0 (0.0)	13 (10.0)	
PD	Yes	58 (38.4)	9 (42.9)	49 (37.7)	0.834
	No	93 (61.6)	12 (57.1)	81 (62.3)	

PVD = posterior vitreous detachment; PD = peripheral degeneration; N = number; *p* = *p*-value, *p* < 0.05; ^1^ some data in these categories were missing from the medical records, and the number of patients for each specific category is lower than the total number of patients; ^2^ retinal tear was not identified preoperatively.

**Table 3:** Distribution of patients according to the type of surgical procedure in relation to the surgical outcome.

			Surgical Outcome		
		Total (N = 151)	Unsuccessful (N = 21)	Successful (N = 130)	*p*
		N (%)			
Type of surgical procedure	Pneumoretinopexy	32 (21.2)	5 (23.8)	27 (20.8)	0.540
	Scleral buckling	13 (8.6)	3 (14.3)	10 (7.7)	
	PPV	106 (70.2)	13 (61.9)	93 (71.5)	
PHACO + PPV	Yes	33 (31.1)	2 (15.4)	31 (33.3)	0.322
	No	73 (68.9)	11 (84.6)	62 (66.7)	

PPV = pars plana vitrectomy; PHACO = phacoemulsification, *p* = *p*-value; N = number; *p* < 0.05.

**Table 4:** Distribution of patients according to postoperative best-corrected visual acuity (BCVA) in relation to preoperative BCVA.

	Preoperative BVCA			*p*
	≤0.1 (N = 108)	>0.1 and <0.5 (N = 17)	≥0.5 (N = 26)	
	N (%)			
Final BCVA				
≤0.1	55 (50.9)	3 (17.6)	2 (7.7)	**<0.001**
>0.1 and <0.5	32 (29.6)	5 (29.4)	2 (7.7)	
≥0.5	21 (19.4)	9 (52.9)	22 (84.6)	

BCVA = best-corrected visual acuity; N = number; *p* = *p*-value, *p* < 0.05.

**Table 5:** Distribution of patients according to the duration of symptoms, macular status, lens status and type of surgery in relation to categories of final best-corrected visual acuity.

			Final BCVA (Decimal)			
		Total	≤0.1	>0.1 and <0.5	≥0.5	*p*
		N (%)				
Symptoms duration (days) ^1^	≤3	42 (29.6)	14 (25.0)	11(29.7)	17 (34.7)	**0.043**
	4–7	37 (26.1)	13 (23.2)	8 (21.6)	16 (32.7)	
	8–14	23 (16.2)	6 (10.7)	8 (21.6)	9 (18.4)	
	15–30	20 (14.1)	8 (14.3)	7 (18.9)	5 (10.2)	
	>30	20 (14.1)	15 (26.8)	3 (8.1)	2 (4.1)	
Macula status ^1^	Attached	63 (42.6)	16 (27.1)	14(36.8)	33 (64.7)	**<0.001**
	Detached	85 (57.4)	43 (72.9)	24(63.2)	18 (35.3)	
Type of surgical procedure	PR	32 (21.2)	6 (10.0)	2 (5.1)	24 (46.2)	**<0.001**
	Scleral buckling	13 (8.6)	0	4 (10.3)	9 (17.3)	
	PPV	106 (70.2)	54 (90.0)	33(84.6)	19 (36.5)	
Lens status	Phakic	94 (62.2)	40 (66.7)	17(43.6)	37 (71)	**0.018**
	Pseudophakic	57 (37.8)	20 (33.3)	22(56.4)	15 (29)	

BCVA = best-corrected visual acuity; PR = pneumoretinopexy; PPV = pars plana vitrectomy; N = number; *p* = *p*-value; *p* < 0.05; ^1^ some data in these categories were missing from the medical records and the number of patients for each specific category is lower than the total number of patients.

**Table 6:** Existing and estimated DRG costs for different surgical procedures.

Surgical Procedure	DRG Cost (Day Surgery (€))	Procedure Time (min)	Cost per min for Day Surgery (€/min)	Estimated DRG Cost (Day Surgery (€))	Inpatient DRG Cost (All Retinal and Cataract Procedures (€))	Cost per min for Inpatient Surgery (€/min)	Estimated DRG Cost (Inpatient Surgery (€))
**PR**	N/A	15	N/A	482.85	1321.63	N/A	396.45
**SB**	N/P	85	N/A	2736.15	1321.63	N/A	2246.55
**PPV**	1609.39	50	32.19	*	1321.63	26.43	***
**PHACO+PPV**	1960.58	62.5	35.38	2211.03 **	1321.63	29.30	1831.01
**PHACO**	601.64	12.5	48.13	*	509.38	40.75	*

PR = pneumoretinopexy; SB = scleral buckling; PPV = pars plana vitrectomy; PHACO = phacoemulsification; N/A = not available; N/P = not practiced; * there is an existing DRG cost for the procedures in question, which is taken for granted in the calculation; ** new estimated cost for the PHACO+PPV procedure was assumed as full costs for the PHACO part in addition to the PPV; *** we assumed that the current inpatient DRG cost for retina procedures (€1321.63) should be the DRG cost for inpatient PPV procedure per se.

**Table 7:** Utility values associated with each surgical outcome and expected QALYs for the treatment.

Surgical Procedure	Surgical Outcome	BCVA	Probability	Utility Value *	Expected QALY	
PR	Unsuccessful	≤0.1	0.096	0.5	0.05	0.11
		≥0.5	0.064	0.9	0.06	
	Successful	≤0.1	0.084	0.5	0.04	0.70
		>0.1 and <0.5	0.084	0.7	0.06	
		≥0.5	0.672	0.9	0.60	
						**0.81**
SB	Unsuccessful	>0.1 and <0.5	0.066	0.5	0.03	0.15
		≥0.5	0.134	0.9	0.12	
	Successful	>0.1 and <0.5	0.24	0.7	0.17	0.67
		≥0.5	0.56	0.9	0.50	
						**0.82**
PPV	Unsuccessful	≤0.1	0.09	0.5	0.05	0.07
		>0.1 and <0.5	0.018	0.7	0.01	
		≥0.5	0.0096	0.9	0.01	
	Successful	≤0.1	0.44	0.5	0.22	0.56
		>0.1 and <0.5	0.264	0.7	0.18	
		≥0.5	0.176	0.9	0.16	
						**0.63**

BCVA = best-corrected visual acuity; PR = pneumoretinopexy; SB = scleral buckling; PPV = pars plana vitrectomy; * averaged values obtained from [25].

## Data Availability

The authors possess all primary data and agree to allow the journal to review the data upon request.
